# The Pedunculopontine Tegmental Nucleus as a Motor and Cognitive Interface between the Cerebellum and Basal Ganglia

**DOI:** 10.3389/fnana.2016.00109

**Published:** 2016-11-07

**Authors:** Fumika Mori, Ken-ichi Okada, Taishin Nomura, Yasushi Kobayashi

**Affiliations:** ^1^Laboratories for Neuroscience Visual Neuroscience Group, Graduate School of Frontier Biosciences, Osaka UniversityOsaka, Japan; ^2^Center for Information and Neural Networks (CiNet), National Institute of Information and Communications Technology and Osaka UniversityOsaka, Japan; ^3^Bio-Dynamics Group, Department of Mechanical Science and Bioengineering, Graduate School of Engineering Science, Osaka UniversityOsaka, Japan; ^4^Research Center for Behavioral Economics, Osaka UniversityOsaka, Japan

**Keywords:** pedunculopontine tegmental nucleus, basal ganglia, cerebellum, Parkinson’s disease, deep brain stimulation

## Abstract

As an important component of ascending activating systems, brainstem cholinergic neurons in the pedunculopontine tegmental nucleus (PPTg) are involved in the regulation of motor control (locomotion, posture and gaze) and cognitive processes (attention, learning and memory). The PPTg is highly interconnected with several regions of the basal ganglia, and one of its key functions is to regulate and relay activity from the basal ganglia. Together, they have been implicated in the motor control system (such as voluntary movement initiation or inhibition), and modulate aspects of executive function (such as motivation). In addition to its intimate connection with the basal ganglia, projections from the PPTg to the cerebellum have been recently reported to synaptically activate the deep cerebellar nuclei. Classically, the cerebellum and basal ganglia were regarded as forming separated anatomical loops that play a distinct functional role in motor and cognitive behavioral control. Here, we suggest that the PPTg may also act as an interface device between the basal ganglia and cerebellum. As such, part of the therapeutic effect of PPTg deep brain stimulation (DBS) to relieve gait freezing and postural instability in advanced Parkinson’s disease (PD) patients might also involve modulation of the cerebellum. We review the anatomical position and role of the PPTg in the pathway of basal ganglia and cerebellum in relation to motor control, cognitive function and PD.

## Introduction

**Figure 1:**
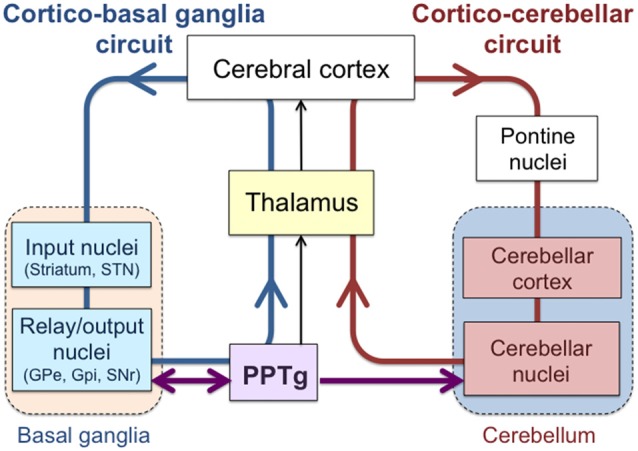
**Interconnections among the cerebral cortex, basal ganglia, cerebellum and pedunculopontine tegmental nucleus (PPTg).** Loops that link the cerebral cortex with the basal ganglia (Cortico-basal ganglia circuit; blue) and with the cerebellum (Cortico-cerebellar circuit; red) are shown. PPTg is intimately connected with the basal ganglia nuclei and also projects to the cerebellar nuclei.

It is conventionally accepted that the cerebellum and basal ganglia belong to segregated systems involved in different features of the functional execution of motor and cognitive behaviors (Middleton and Strick, 2000). The cerebellum has been regarded as a locus that contributes to flexible modification of behavior and error-based learning (Wolpert et al., 1998; Ito, 2002), whereas the basal ganglia is considered to play a role in reward prediction and reward-based learning (Doya, 2000; Houk, 2005). Anatomically, the loop that connects the cerebellum and the cerebral cortex is separated from that between the basal ganglia and cerebral cortex (Figure 1; Middleton and Strick, 2000; Graybiel, 2005). Signals from the cerebellum and basal ganglia are relayed via different thalamic nuclei and then project to the cerebral cortex (Percheron et al., 1996; Sakai et al., 1996), which is considered to be an interface between those two systems.

However, in a recent series of studies, Strick and his colleagues reported the existence of two disynaptic pathways that connect the cerebellum and basal ganglia by using novel trans-neuronal transport of the rabies virus in monkeys (Bostan and Strick, 2010). One links the dentate nucleus with the striatum through thalamic nuclei (Hoshi et al., 2005), whereas the other connects the subthalamic nucleus (STN) with the cerebellar cortex via the pontine nuclei (Bostan et al., 2010). The defining of these pathways promoted understanding of the role of connections between the cerebellum and basal ganglia in motor disorders. In the 1960s, early attempts were made to alleviate dyskinesia by conducting lesions in the dentate nucleus (Higgins and Glaser, 1965; Heimburger, 1967; Zervas et al., 1967, 1968). Parkinson’s disease (PD) is a neurodegenerative disorder caused mainly by dysfunctions of the dopaminergic system in the basal ganglia (Pahapill and Lozano, 2000), whereas pathophysiological changes were also reported in other brain regions including cerebellum (Wu and Hallett, 2013). In PD patients, increased and oscillatory activities of the STN and hyperactivation of the cerebellum were observed (Rascol et al., 1997; Yu et al., 2007; Amtage et al., 2008). Furthermore, deep brain stimulation (DBS) of the STN normalized cerebellar activity and reduced their motor disorders (Limousin-Dowsey et al., 1999; Grafton et al., 2006), possibly by a STN-cerebellum pathway (Bostan and Strick, 2010).

The pedunculopontine tegmental nucleus (PPTg, also known as PPTN or PPN) of the brainstem, which intimately connects with the basal ganglia, was recently reported to project fibers to the cerebellum and to synaptically activate the deep cerebellar nuclei (Vitale et al., 2016). This raises the possibility that the PPTg and basal ganglia relay reward and motivational information (Keating and Winn, 2002; Okada et al., 2009) and modulates cerebellum activity and motor output signal. It was also reported that DBS of the PPTg in a PD patients could induce a therapeutic effect for symptoms that were refractory to dopaminergic treatment (Ferraye et al., 2010; Moro et al., 2010). In this mini review article, we address the classical view of the PPTg and its position within the cerebral cortex-basal ganglia-brainstem-cerebellum circuit in the context of motor control, reward and cognitive function.

## PPTg as a Part of Basal Ganglia

The PPTg is a rostral brainstem nucleus located in the pontomesencephalic reticular formation and adjacent to the superior cerebellar peduncle. Mesulam et al. (1983) classified cholinergic neurons in the PPTg that project to the thalamus as Ch5 group (Mesulam et al., 1983). The PPTg also contains non-cholinergic neurons such as glutamatergic and GABAergic neurons (Jones and Beaudet, 1987; Clements and Grant, 1990; Spann and Grofova, 1992; Ford et al., 1995; Wang and Morales, 2009). A more recent report suggests that there is a different intrinsic connectivity in the rostral vs. caudal aspects of the PPTg, but the functional relevance remains unknown (Martinez-Gonzalez et al., 2011). In addition to its ascending projection to the thalamus, the PPTg is also connected with the basal ganglia (Edley and Graybiel, 1983), cerebral cortex (Woolf and Butcher, 1989) and the brainstem reticulospinal tract (Rye et al., 1988).

**Figure 2:**
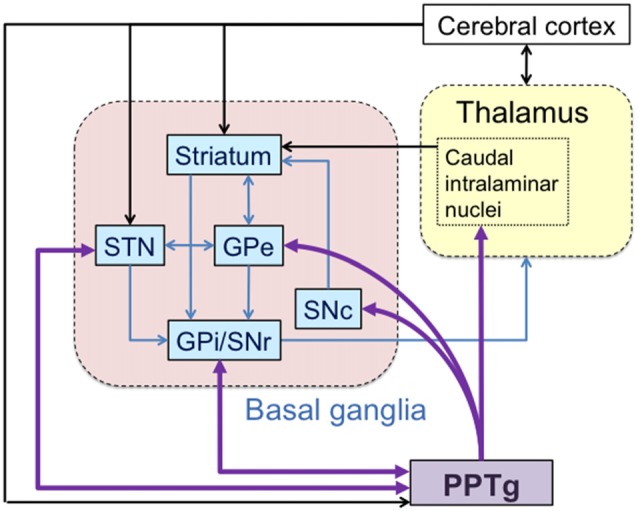
**Detailed connections between the PPTg and basal ganglia.** The basal ganglia forms several internal circuits. The PPTg have reciprocal connections with the subthalamic nucleus (STN), internal globus pallidus (GPi) and substantia nigra pars reticulata (SNr). The PPTg also projects to the external globus pallidus (GPe), substantia nigra pars compacta (SNc) and centromedian nucleus (CM).

Among these connections, the PPTg establishes unique reciprocity with the basal ganglia (Figure 2; Mena-Segovia et al., 2004). The output nuclei of the basal ganglia, the substantia nigra pars reticulata (SNr) and internal globus pallidus (GPi), are reciprocally connected with the PPTg (Moriizumi and Hattori, 1992; Semba and Fibiger, 1992; Groenewegen et al., 1993). SNr projections to the PPTg are GABAergic and decrease the activation of cholinergic (Kang and Kitai, 1990; Granata and Kitai, 1991; Saitoh et al., 2003) and non-cholinergic (Saitoh et al., 2003) PPTg neurons. Mixed cholinergic and glutamatergic projections then return from the PPTg to the SNr and GPi and also to the external globus pallidus (GPe; Clarke et al., 1987, 1997; Charara and Parent, 1994; Lavoie and Parent, 1994). The STN innervates the PPTg through glutamatergic projections and the PPTg sends both glutamatergic and GABAergic projections back to the STN (Hammond et al., 1983; Bevan and Bolam, 1995). The PPTg projects to the dopaminergic neurons in the substantia nigra pars compacta (SNc; Jackson and Crossman, 1983; Beninato and Spencer, 1987), these neurons are thought to play an important role in the reinforcement learning (Alderson et al., 2006; Winn, 2006; Wilson et al., 2009). Cholinergic and glutamatergic neurons in the PPTg make synaptic connections with dopaminergic neurons (Scarnati et al., 1986; Futami et al., 1995; Takakusaki et al., 1996). Electrical stimulation of the PPTg induces a burst firing of dopaminergic neurons (Lokwan et al., 1999; Floresco et al., 2003), and induces the release of dopamine in the striatum (Chapman et al., 1997; Forster and Blaha, 2003; Miller and Blaha, 2004). The PPTg also controls activity of the striatum through its bilateral projection to the caudal intralaminar nuclei (Erro et al., 1999; Barroso-Chinea et al., 2011).

## PPTg and Basal Ganglia as a Limbic-Motor Interface

Classically, the PPTg is thought to be involved in locomotion and wake-sleep cycles. Several of its motor and non-motor features are the subjects of previous reviews (Inglis and Winn, 1995; Takakusaki, 2009; Garcia-Rill, 2015).

The PPTg is included in the mesencephalic locomotor region (MLR) that regulates walking and running (Skinner and Garcia-Rill, 1984; Sherman et al., 2015). The MLR was first discovered in the cat and then in various vertebrate species from lampreys to monkeys. Various human brainstem nuclei, specifically the PPTg, cuneiform, and subcuneiform nuclei, exhibited increased activity when participants were asked to imagine that they were walking, and these have been found to correspond to the MLR (Tattersall et al., 2014). The PPTg is regarded as being involved in locomotion, while the anatomical contribution of the PPTg to the MLR is still controversial, but it is regarded as being involved in locomotion, which is one of the main functions of MLR (Ryczko and Dubuc, 2013; Sloan et al., 2015). As low-threshold electrical stimulation of the PPTg induces locomotion (Garcia-Rill et al., 1987), it has been accepted that the PPTg acts as a relay from the basal ganglia to the spinal cord (Takakusaki et al., 2004a). However, the PPTg is regarded as more than a simple relay (Mena-Segovia et al., 2004), based on new studies showing that it also controls postural muscle tone (Takakusaki et al., 2004a) and saccades (Kobayashi et al., 2004). Certainly, the basal ganglia, PPTg, and spinal cord all have common functions that are beyond motor control, and are mutually influenced to some extent (Takakusaki et al., 2004a).

The PPTg, together with the brainstem cholinergic laterodorsal tegmental nucleus, is located in a rostral area between the mesencephalon and the centrum semiovale (Rothballer, 1956); forms the ascending reticular activating system (RAS) that connects the brainstem to the cortex, and controls cognitive processes such as attention, learning, memory, wakefulness, and sleep-wake transitions (Garcia-Rill, 2015). The RAS is modulated by both acetylcholine (ACh) and adrenaline, which work together and also competitively to control thalamocortical activity and corresponding behavioral states. PPTg neurons are active during waking and rapid eye movement sleep (Garcia-Rill et al., 2007). Cholinergic activation in the RAS leads to increased ACh release throughout the reticular formation as well as in the substantia nigra, basal forebrain, thalamus, and cerebellum (Garcia-Rill, 1997). The basal ganglia are also involved in regulating the sleep-wake cycle (Mena-Segovia et al., 2002), and its reciprocal relationships with the PPTg are crucial for this function (Mena-Segovia and Giordano, 2003; Takakusaki et al., 2004c). Garcia-Rill et al. (2007) reported that posterior PPTg neurons are electrically coupled, and that this coupling achieves better spatial summation in the absence of a synaptic time delay. The coordinate rhythmic firing of PPTg neurons and their electrical coupling might underlie and enhance attentional state for tasks such as radial maze (Dellu et al., 1991; Taylor et al., 2004) and eye movement task (Okada and Kobayashi, 2015). As a result, the PPTg promotes sleep-wake transitions as a part of the RAS, and works a crucial element in the generation and maintenance of the rapid rhythms in the cortex, (which are associated with wakefulness and REM sleep).

Moreover, the PPTg has collateral functions with the basal ganglia in attention, reward and learning behaviors. Our group and others have examined neuronal activity of the monkey PPTg under various behavioral conditions. These neurons showed movement-related activity modulation associated with the arm (Matsumura et al., 1997) and eyes (Kobayashi et al., 2002; Okada and Kobayashi, 2009; Hong and Hikosaka, 2014), and are also modulated by arousal levels, task performance and reward (Kobayashi et al., 2002; Okada et al., 2009; Okada and Kobayashi, 2013; Hong and Hikosaka, 2014). Moreover, many neurons exhibited combinations of these multi-modal activities. Furthermore, it is hypothesized that the PPTg is involved in reward prediction error computation, and thus contributes to decision-making (Kobayashi and Okada, 2007). Thus, the PPTg is considered to take part in the facilitation of exogenous sensory processing and central processing for motor commands, by modulating awareness and attentive states via dopaminergic systems (Kobayashi et al., 2002; Takakusaki et al., 2004b).

Therefore, we can hypothesize that the PPTg and basal ganglia play a common role as a limbic-motor interface (Winn et al., 1997; Inglis et al., 2000). As a point of fact, the multiple functions of the PPTg are partially in charge of reward prediction during learning (Brown et al., 1999), which is a function traditionally correlated with the basal ganglia.

## PPTg Projection to the Cerebellum

In addition to its dense connections with the basal ganglia, the PPTg also projects to the cerebellum. Projections from the PPTg have been anatomically identified to the deep cerebellar nucleus in rats (Woolf and Butcher, 1989; Newman and Ginsberg, 1992; Ruggiero et al., 1997). Furthermore, an imaging study has reported this PPTg-cerebellum connection in humans (Aravamuthan et al., 2007), but the function of this connection remained unknown.

Recently, the functional connectivity between the PPTg and deep cerebellar nuclei was investigated by electrical microstimulation of rat PPTg (Vitale et al., 2016). Single pulse microstimulation of the PPTg evokes a brief activation of the deep cerebellar nuclei with a short latency, which suggests that this orthodromic response might be mediated by a direct PPTg-cerebellar excitatory pathway. The dentate nucleus has the highest rate of recorded neurons that responded to PPTg stimulation, while a lower extent of neurons was activated in the fastigial and interpositus nucleus. Using an iontophoretic approach with ACh antagonists, Vitale et al. (2016). further demonstrated the involvement of ACh in the evoked responses of cerebellar neurons. Also, only a small number of antidromic responses were recorded in the PPTg. This is consistent with the original anatomical report in cats that did not find projections from the cerebellar nuclei to the PPTg (Edley and Graybiel, 1983). Although there is a species difference such that fibers from the deep cerebellar nuclei directed to the PPTg in monkeys (Hazrati and Parent, 1992).

This raises the possibility that the PPTg acts as an interface between the cerebellum and basal ganglia to influence motor control and cognitive functions. Classical studies postulate that the cerebellum plays a pivotal role in adaptive behavioral control (Ito, 2002). Stiffness control, at the ankles for instance, is required for locomotion and stabilizing posture. However, large stiffness yield by large neural feedback gains would easily induce instability due to its feedback delay. Such instability can be compensated by phasic modulation of feedback gains, as hypothesized by intermittent control (Suzuki et al., 2012). Interestingly, phasic or intermittent control of feedback gains can be established through reinforcement learning with a simple reward function (Michimoto et al., 2016). The PPTg might relay reward and reinforcement learning information from the basal ganglia to the cerebellum, and might play a crucial role in generating and regulating postural tonus and stabilizing posture. Thus, dysfunction of the basal ganglia in patients with PD might induce postural instability (Suzuki et al., 2012).

## Therapeutic Effect of PPTg Stimulation in Parkinson’S Disease

Recent advances in PD therapy using DBS of the PPTg provide a hint of its involvement in coordinating the function of the basal ganglia and cerebellum. PD patients show a variety of motor and non-motor impairment, including PPTg-related-symptoms, in particular, locomotor abnormalities such as shorter steps and slowness of walking, and also RAS-related deficits such as arousal and hyperactive reflexes. Anatomically, cholinergic and glutamatergic excitatory projections from the PPTg regulate activity of dopaminergic neurons in the SNc and VTA (Takakusaki et al., 1996). These observations suggest that the PPTg is related to PD (Garcia-Rill, 2015).

More specifically, dysfunction within the PPTg leads to various PD-like movement disabilities. For example, inhibition of neurons in the PPTg delayed movement onset and slowed the acceleration as well as the deceleration and amount of arm movements in primates (Matsumura and Kojima, 2001). Also, unilateral PPTg lesions in monkeys led to hemiparkinsonism in the contralateral side (Kojima et al., 1997). These reports support a facilitatory function by the PPTg of spontaneous extremity movements through its excitatory projections to the dopaminergic neurons (Kojima et al., 1997).

While PD is thought to be a dopaminergic disorder, many studies reported that freezing of gait and postural instability, both symptoms of advanced PD, are resistant to dopaminergic medication (Ferraye et al., 2010; Moro et al., 2010). Studies of PD patients and monkeys demonstrated that the severities of gait and posture impairments were correlated with the extent of ACh neuronal loss in the PPTg (Karachi et al., 2010). DBS of the PPTg has emerged as an effective treatment for the symptoms like freezing of gait and postural instability that are refractory to dopaminergic medication (Ferraye et al., 2010; Moro et al., 2010). One hypothetical pathway suggested by these results is that gait and axial disturbances are attributed to a disruption of ACh mechanisms in the brainstem, and effects of DBS might be obtained through surviving ACh fibers projecting from the PPTg to cerebellum (Aravamuthan et al., 2007) and then relayed via the cerebello-thalamo-cortex pathway. In PD patients exhibiting a freezing of gait, alpha range power (7–12 Hz) of local field potentials of the PPTg was correlated with gait speed and the power attenuated with gait freezing (Thevathasan et al., 2012). This oscillatory activity in the PPTg was also reported in healthy animals (Mena-Segovia et al., 2008; Okada and Kobayashi, 2015). Taken together, these reports might suggest that rhythmic activity is a feature of the functioning PPTg, which may change according to behavioral condition.

In addition to axial movement disorders, PD patients also exhibit eye movement control disabilities, especially in saccades. The saccadic eye movement system is one of the most well-studied systems in the brain. Saccades are regulated across a distributed network of the brain, including the cerebral cortex, basal ganglia, and cerebellum, and are executed by the well-understood brainstem circuitry (Munoz and Fecteau, 2002). Neuronal activity in the basal ganglia is modulated by expected reward, and plays a key role in guiding the eyes to the location where reward is available (Hikosaka et al., 2006). On the other hand, neurons in the posterior vermis, caudal fastigial nucleus, and interpositus nucleus of the cerebellum are related to the precise and adaptive control of saccades (Robinson and Fuchs, 2001; Dash and Thier, 2014). Recent studies reported that single neurons in the oculomotor vermis and caudal fastigial nucleus discharged both for macro- and micro-saccades (Arnstein et al., 2015; Sun et al., 2016). We previously reported that neurons in the monkey PPTg showed saccade-related activity modulation, some modulations were only associated with reward-related saccades consistent with neurons reported in the basal ganglia (Kobayashi et al., 2002; Okada and Kobayashi, 2009), while others exhibited this modulation with every saccade, including small fixational saccades, consistent with neurons in the cerebellum (Okada and Kobayashi, 2014). One possibility is that the PPTg acts as an interface between the basal ganglia and cerebellum, and thus reward and cognitive signals influence precise microsaccades (Joshua et al., 2015; Yu et al., 2016). Analyzing saccade-associated neuronal activity may be a key tool for understanding the roles of PPTg within the cerebellar and basal ganglia networks in health and disease conditions.

## Conclusion

Based on traditional knowledge about the functions and connections of the PPTg, and given the existence of fibers from the PPTg to the cerebellum, we suggest that the PPTg acts as an interface between the basal ganglia and cerebellum, thereby influencing motor control and cognitive functions. From this standpoint, the therapeutic effects of PPTg DBS in patients with PD insensitive to dopamine treatment might occur via transmission of the artificial signal to the cerebellum. Thus, it is likely that the PPTg has a role in monitoring the balance between basal ganglia and cerebellum, and thereby controlling cerebral activity.

## Author Contributions

FM, KO, TN and YK wrote the article.

## Conflict of Interest Statement

The authors declare that the research was conducted in the absence of any commercial or financial relationships that could be construed as a potential conflict of interest.

## Abbreviations

Ach: Acetylcholine
DBS: Deep brain stimulation
MLR: Mesencephalic locomotor region
PD: Parkinson’s disease
PPTg: Pedunculopontine tegmental nucleus
RAS: Reticular activating system.
